# Analyzing the Effects of Calcium Nitrate over White Portland Cement: A Multi-Scale Approach

**DOI:** 10.3390/ma16010371

**Published:** 2022-12-30

**Authors:** Mihai Marius Rusu, Adriana Vulpoi, Cristian Vilau, Cristian Mircea Dudescu, Petru Păşcuţă, Ioan Ardelean

**Affiliations:** 1 Department of Physics and Chemistry, Technical University of Cluj-Napoca, 400114 Cluj-Napoca, Romania; 2 Nanostructured Materials and Bio-Nano-Interfaces Center, Institute of Interdisciplinary Research in Bio-Nano-Sciences, Babes-Bolyai University, T. Laurean 42, 400271 Cluj-Napoca, Romania; 3 Department of Mechanical Engineering, Technical University of Cluj-Napoca, 400114 Cluj-Napoca, Romania

**Keywords:** calcium nitrate tetrahydrate, white Portland cement, cement paste hardening, microstructure, electron microscopy, NMR relaxometry

## Abstract

Calcium nitrate is considered a promising accelerator in cement-based composites, with high potential in 3D printing and cold cement concreting. The effect induced by the composition of calcium nitrate tetrahydrate (CN) accelerator into white Portland cement is evaluated here from three perspectives: (1) Fresh cement paste properties in terms of setting time and slump, (2) mechanical properties of hardened cement samples at 7 and 28 days and (3) material characteristics in terms of structure and porosity that further link the presence of the accelerator with the macroscopic performances. The compressive and flexural strength of the hardened samples, evaluated after 7 and 28 days of hydration, indicate a non-monotonous trend with CN concentration. Crystalline phase composition is investigated using X-ray diffraction (XRD). The morphology and texture are analyzed at the flexure interface by visual inspection and electron microscopy. Complementary, the porous features are investigated by NMR-relaxometry on dry and cyclohexane-filled samples. The studies confirm that CN promotes changes in the composition and morphology of hydrates, while a trend of increase in capillary porosity is outlined as well. This competition between multiscale effects may be quantified by NMR and complementary techniques to further clarify the mechanical behavior of such composites.

## 1. Introduction

There is an ongoing search for more environmentally friendly and cost-efficient construction technologies. A growing interest concerns additive manufacturing and other 3D printing techniques as they allow further automation and lower time spans. They also reduce the environmental impact by efficient management of building materials and favor more complex architectural designs [1,2]. However, the composites ideal for 3D printing require a challenging balance between pumpability, extrudability on one side and workability and fast structural build-up on the other. Hence, ongoing research focuses on the role played by various admixtures, including viscosity modifying agents, superplasticizers, pozzolans and accelerators in tuning the rheology, setting time and strength development of cementitious materials [3,4].

Chemical accelerators can be introduced via pre-mixing or inline mixing when a fast structural build-up is required, i.e., for rapid repair works, concrete spraying, and extrusion-based 3D printing [5]. Their major role is to modify the hydration rate of the primary cementitious particles so that setting and/or hardening may take place earlier. In this context, setting accelerators mainly interfere with the reaction between tricalcium aluminate (C_3_A) and gypsum (CS), leading to a faster C_3_A hydration [5,6], while hardening accelerators affect alite (C_3_S) hydration and the formation of calcium-silicate-hydrate (C-S-H) [5]. Some of the most noteworthy accelerating admixtures, both for set and hardening processes, include CaCl_2_, Ca(NO_2_)_2_, and Ca(NO_3_)_2_ [7]. Even though extensive research describes the effects induced by the most efficient CaCl_2_ accelerator [8], further studies are required for other compounds, including Ca(NO_3_)_2_ (denoted here as CN). CN is viewed as a commercially available accelerator exhibiting other notable functionalities for cold weather concreting [9,10] and for corrosion inhibition [11]. The accelerating mechanisms resemble the ones proposed for CaCl_2_: (1) CN affects early C_3_A hydration, possibly by incorporating (NO_3_)_2_^+^ into AFm structures and inhibiting ettringite (AFt) and monosulfoaluminate (m-AFm) growth [7,12] and (2) CN may promote C_3_S and C_2_S hydration due to an increase in portlandite (CH) contributions at earlier stages [5,7]. During parallel studies on the effects of CN dosage on the mechanical properties of hardened cements, it was observed that intermediate CN concentrations might increase the compressive strength of different cementitious systems [7,10]. In the early work, Justnes et al. [13] relate higher CN-induced compressive strength with modifications in morphology and composition of C_3_S hydrates (i.e., higher C-S-H gel strength potential), while their later studies noted some CN-induced changes to porosity as well [10].

In our recent papers, we focused on NMR studies of white Portland cement-CN composites during early hydration stages [14,15] and the deceleration stage at 7 and 28 days [15]. It was observed that the presence of CN induced an increase in the fractal dimension of the porous systems [14], and it can be further hypothesized that the CN acceleration is linked with variations in the growth patterns of C-S-H and the interference with C_3_A-CS reaction, that may lead to inhibited ettringite growth and/or variations in AFm structure [6]. Further on, white cement can be distinguished from ordinary grey cement by having a lower content of iron and manganese oxide. Due to this feature, lower internal gradient effects are expected on echo attenuation in CPMG relaxation measurements as compared with grey cement. This allows reliable comparison of the information extracted via NMR relaxometry and the mechanical strength tests. In the present work, we complement the previous findings with insight into the effect induced by CN at several concentrations in white Portland cements from the perspective of: (1) The fresh paste properties, (2) both compressive and flexural strength development (at 7 and 28 days), and (3) morphological and structural data analysis in terms of mineralogic composition, microstructure (multi-scale analysis of the flexure plane after 28 days), and porosity evaluation through various NMR relaxometry experiments performed on samples after flexure tests. The NMR experiments were done on vacuum-dried samples as well as on the same media filled with cyclohexane. As described in previous studies, the use of cyclohexane in later experiments represents a relatively new approach that enables better discrimination between the different pore classes found in cement [15,16,17].

## 2. Materials and Methods

### Reagents

The cement pastes were based on the following reagents: White Portland cement (CEM I 52.5 R, Holcim, Bucharest, Romania), tap water (stored at room temperature), and calcium nitrate (Ca(NO_3_)_2_ tetrahydrate, NORDIC Chemicals SRL, Cluj-Napoca, Romania). A water to cement mass ratio of 0.4 was maintained for all experiments. The CN accelerator to cement mass ratio varied between 0.00 and 0.03, to yield samples CPAx where “x” stands for 0.0x in the accelerator to cement ratio. Initially, CN was dissolved in the entire water volume to be used. The obtained solutions were added to the cement mass, followed by a 2 min paste homogenization with a mixer at constant shear rates. The samples were prepared under normal room temperature (~20 °C) and humidity conditions (~40%) and stored for curing in plastic containers.

Slump tests were performed using a miniaturized slump cone (inner diameters of 22 and 37 mm and a 58 mm height) [18]. The cone was filled with fresh cement paste immediately after the mixing step and cast on a glass support. The cone was carefully lifted in a single vertical movement to avoid any inertial effects. The slump (S) and the degree of spread (R) of the pastes were determined under equilibrium conditions. The procedure was repeated 3 times to evaluate the repeatability of the experiment. 

The initial and final setting time measurements were performed using a Vicat instrument (Paris, France) in accordance with ASTM C 191-04a standards. Fresh paste compositions were poured into Vicat containers immediately after mixing. To determine the initial setting time, the experimental data were fitted with a sigmoidal curve using the Origin software.

In order to evaluate the mechanical properties of the hardened cement samples (after 7 and 28 days from initial hydration), mechanical compression and bending tests were performed. The compression tests were performed on cubic specimens of 20 mm side length, using the INSTRON 8801–100 KN equipment (Norwood, MA, USA), with a maximum force of 100 KN (10 tons force). The bending tests were performed on 80 × 20 × 20 mm specimens, using the INSTRON 3366–10 KN equipment, with a maximum force of 10 KN (1 ton force). Both compression and bending tests were carried out until sample failure was reached.

After bending tests, the samples were inspected using a Canon EOS 750D camera (Tokyo, Japan) equipped with a Sigma DG MACRO HSM lens (Kawasaki, Japan). The microstructure of the fracture plane exposed by flexure was further investigated using scanning electron microscopy (SEM). Fragments of the cement samples were fixed on a carbon film attached to the sample support. To minimize the surface charging effects, the samples were coated with a thin Au layer. The samples were then analyzed using a Dual Beam FEI Quanta 3D FEG electron microscope with DET detector (Everhart Thornley detector). Multiple regions (monolith core, interface with the surface, embedded voids, etc.) of each cement-accelerator system have been analyzed at different scales. Panoramic images obtained from samples CPA0 and CPA2 by stitching 2000x micrographs were further processed and analyzed using FIJI software [19] to differentiate the macropore (capillary pore) networks.

Powder X-ray diffraction measurements (XRD) were performed at room temperature using an XRD-6000 Shimadzu diffractometer. The measurements were performed with an X-ray tube (Cu-K_α_, λ= 1.5406 Å). A graphite monochromator was mounted on the detector arm. The diffractograms were recorded in the range between 2θ = [5, 70°] at a voltage of 40 kV and a current of 30 mA, with a speed of 2°/min and a step of 0.02°. The cement samples resulted after a hydration time of 7 days and 28 days were ground and mounted on glass supports. To evaluate any accelerator induced changes in mineral composition, a quantitative crystalline phase analysis was performed using the Material Analysis Using Diffraction (MAUD) software [20].

NMR relaxometry investigations were performed on the monolithic samples after flexure tests. A Bruker Minispec MQ20 instrument (Bruker BioSpin GmbH, Rheinstetten, Germany) was operated at a proton resonance frequency of 20 MHz. Cyclohexane was selected as a probing molecule, as described elsewhere [16]. The samples were further dried in a vacuum oven at 80 mbar and 60 °C for 30 min to extract the adsorbed water. Then, the samples were soaked in cyclohexane to saturate the porous structure of the cements. Prior to measurements, the samples were extracted from the bulk solution and inserted into 10 mm measuring tubes. The transverse relaxation time (T_2_) distributions of the molecules confined inside the porous media were determined using the Carr–Purcell–Meiboom–Gill (CPMG) technique [21]. To reduce diffusion effects in the internal gradients, CPMG echo trains comprising 1000 echoes, with echo time intervals of 80 µs and a recycle delay of 5 s were detected. A total number of 128 measurements were averaged to increase the signal to noise ratio. The measurements were performed at a constant temperature of 35 °C. The relaxation time decay signals were further processed using inverse Laplace transformation algorithms [22]. The resulting T_2_ distributions were finally ascribed with the size distribution of different pore classes as consistent with previous works [15,16].

## 3. Results

### 3.1. Characterization of Fresh Pastes 

**Figure 1:**
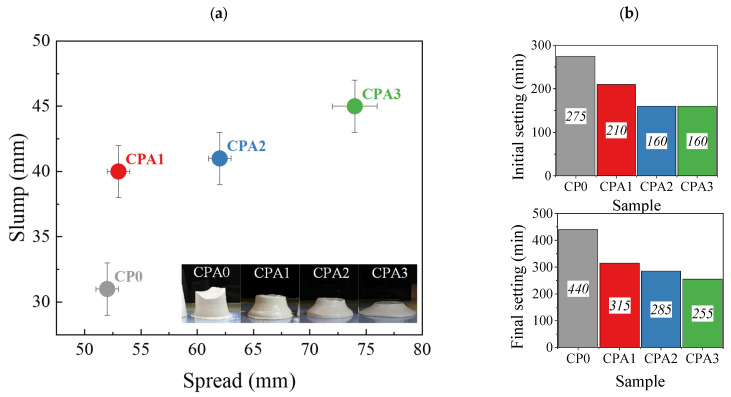
Fresh CPA paste properties: (**a**) Mini-slump tests giving the average height and spread of the casted cement samples (see inset) and (**b**) initial and final setting times determined using the Vicat needle test.

The fresh paste properties in terms of slump and setting time are shown in Figure 1. The averaged slump and spread of the cement pastes for each paste composition are shown in Figure 1a. The data indicate their progressive increase with CN content. These variations are correlated with the change of the rheological parameters, the flowability, and early strength development of the modified cement pastes and are consistent with the literature. For instance, when investigating the rheology and buildability of ordinary Portland cements with CN, Souza et al. [3] revealed increased flowability (lower yield stress values) during the first 20 min for 1% and 2% CN compositions and the opposite for 5% CN compositions. Skripkiunas et al. [10] obtained a minimum in the dynamic viscosity of fresh cement-CN pastes for 1.5% CN, while concentrations up to 3% CN led to comparable or higher viscosities with respect to the reference sample. The early-age behavior modified with increasing accelerator content (i.e., CaCl_2_, CN, etc.) was interpreted by considering the mechanisms of set and hardening acceleration: (1) A delayed AFt formation at lower accelerator content, (2) the hydration of C_3_S leading to faster C-S-H precipitation and also (3) pH changes and (4) enhanced coagulation of Ca(OH)_2_/hydroxysilicates [3]. Indeed, at higher CN weight percentages, Yuan et al. [4] have observed not only a fast structural build-up and hydration heat release (maximum heat release at about 5–2 h for CN1-2 versus 6 h for reference sample) but also higher pH, zeta potentials and ionic conductivities for higher CN concentrations. A rapid increase in pH values was observed during the first 15 min, while a linear increase in zeta potential and conductivity were obtained for longer hydration times [4].

The same mechanisms need to be analyzed when the hydration products form an interconnected network and the setting of the cement paste occurs. The efficiency of CN as a set accelerator is reflected by the initial and final Vicat setting times presented for different compositions in Figure 1b. The CPA0 sample exhibited an initial setting time of 275 min and a final setting time of 440 min. The values reduce monotonously following the increase in CN amount in the given concentration range. The initial setting time will be reduced to 76% with respect to the control sample for CPA1 and will stabilize at ~58% for CPA2 and CPA3 samples. The final setting time follows a similar path. The relations between CN concentration and the setting time of different types of cement are analyzed in multiple studies and reviews [7], while some reveal a monotonous or complex relation between the two parameters [9,10], the present trend is consistent with the studies performed by Dorn et al. [23], that also revealed faster C_3_S consumption, and growth of portlandite (CH) during early stages.

### 3.2. Structural Analysis of Hardened Pastes

**Figure 2:**
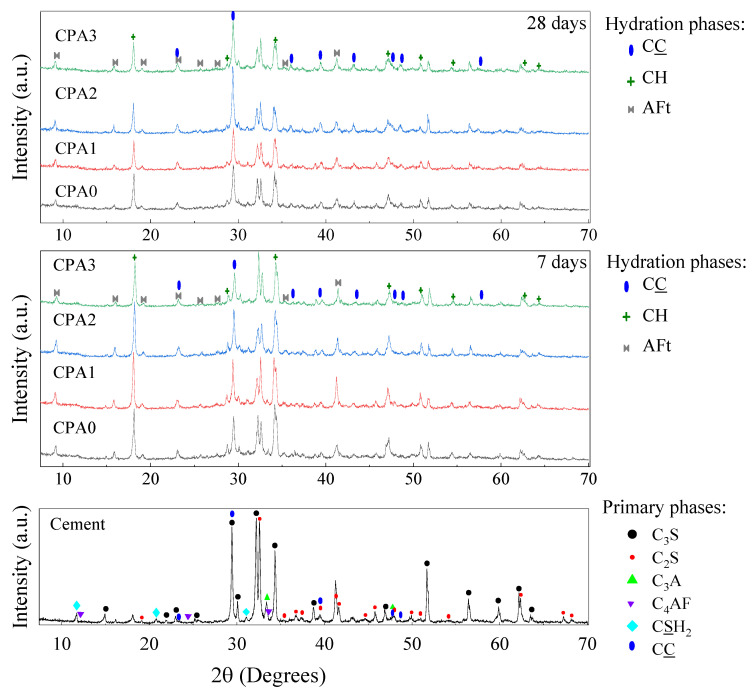
X-ray diffraction patterns for cement (no hydration) and CPA samples after hydration at 7 days and 28 days together with the identified crystalline phases: Alite (C_3_S), belite (C_2_S), gypsum (CS), tricalcium aluminate (C_3_A) and aluminoferite (C_4_AF). Hydration compounds: Portlandite (CH), calcite (CC), and ettringite (AFt).

The diffractograms obtained for the cement samples with different accelerator ratios are shown in Figure 2. Before the hydration step, the main signals observed in the cement sample correspond to a crystalline phase composition based on tricalcium silicate (alite, further denoted C_3_S), dicalcium silicate (belite, C_2_S), and gypsum impurities (CaSO_4_∙2H_2_O, denoted as CS).

**Figure 3:**
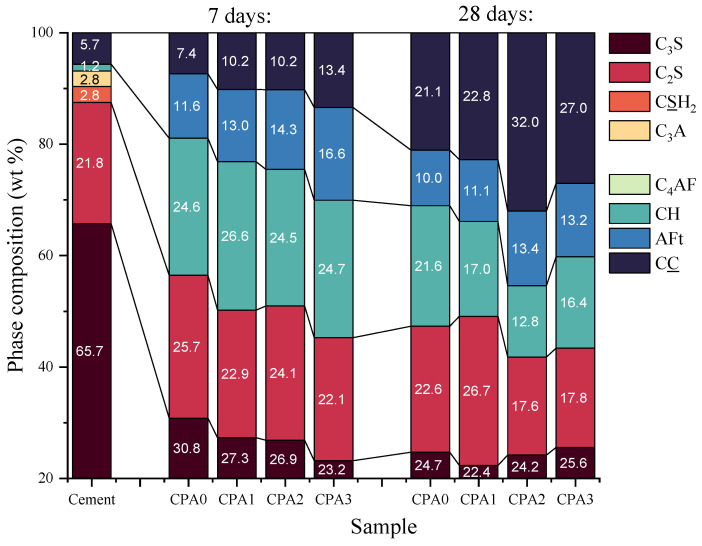
Results of the quantitative crystalline phase analysis using the XRD patterns for cement and CPA composites after hydration at 7 and 28 days.

During the Rietveld analysis [24], tricalcium aluminate (Ca_3_Al_2_O_6_, denoted C_3_A), tetracalcium aluminoferrite (C_4_AF), portlandite (Ca(OH)_2_, denoted as CH) and carbonate (CaCO_3_, denoted CC) were also included. After curing the samples for 7 and 28 days, the diffractograms trace the crystalline phase composition based on the unhydrated C_3_S and C_2_S remnant phases in parallel with the developing hydration products: Ettringite (denoted AFt), CH, and CC. One can observe a change in the relative intensities of the main signals 2θ = 30° relative to 2θ = 32°. As reflected by the crystalline phase composition (Figure 3), the changes are associated with the development of AFt and CH phases and the progressive consumption of C_2_S and C_3_S.

Following the increase in CN concentration, high variations are observed mainly in CC and CH contributions. The changes are mainly attributed to carbonation of CH and perhaps other amorphous phases, more pronounced at a higher CN dosage. The higher CC content obtained for samples CPA2 and CPA3 after 28 days of hardening may indicate the first signs of higher porosity. However, the carbonation front depends upon several parameters, including the porosity, relative humidity, CO_2_ diffusivity, and the formation of carbonatable products, including CH and C-S-H. This aspect requires further insights and should be addressed separately in future studies.

### 3.3. Mechanical Properties of Hardened Pastes

**Figure 4:**
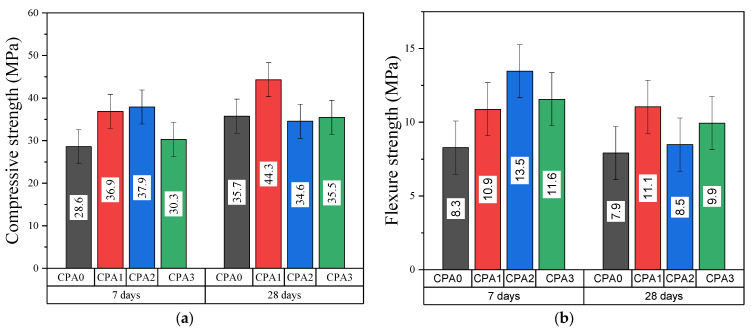
Mechanical properties of CPA samples at 7 and 28 days: (**a**) Compressive strength and (**b**) flexural strength.

The results of mechanical tests performed at 7 and 28 days after hydration are shown in Figure 4. Similar trends are observed for the dependence of compressive and flexural strength with CN addition. Both compressive and flexural tests indicate that the CN addition tends towards improving the strength of the cement paste but at an optimum amount. A non-monotonous increase in compressive and flexural strengths with CN addition is best represented for the tests performed after 7 days of hydration. As observed in Figure 4a, the compressive strength values for the CPA1 and CPA2 are 37 and 38 MPa, respectively, compared to that of 29 MPa for the control sample. After 28 days of hydration, the compressive strength tends to increase: Sample CPA1 exhibits the highest value of 44 MPa versus 35 MPa obtained for the test sample. At a higher CN concentration, the compressive strength decreases at 35 MPa for sample CPA2, while only a minute increase is obtained for CPA3.

Similar trends were observed by Skripkiunas et al. [10] for concretes based on CEM IIR and CN cured at 20 °C, and they further attributed this behavior to changes in porosity. They successfully demonstrated that CN can promote even higher compressive strengths when curing the samples at 5 °C. However, the effect of CN concentration on the porosity and the mechanical strength needs further in-depth investigations. Now, considering the flexure tests (Figure 4b) at higher CN values, the flexural strength tends to decrease from 7 to 28 days of hydration. After 7 days, sample CPA2 exhibits the highest value of 13 MPa versus 8 for CPA0, while again, after 28 days of hydration, the highest strength reaching 11 MPa is obtained for sample CPA1, compared with 8 MPa for CPA0. At a higher CN concentration, the flexure strength decreases to 8 MPa for CPA2. A slight increase to intermediate 10 MPa is finally reached for CPA3.

### 3.4. Correlations with Structural and Microstructural Features

**Figure 5:**
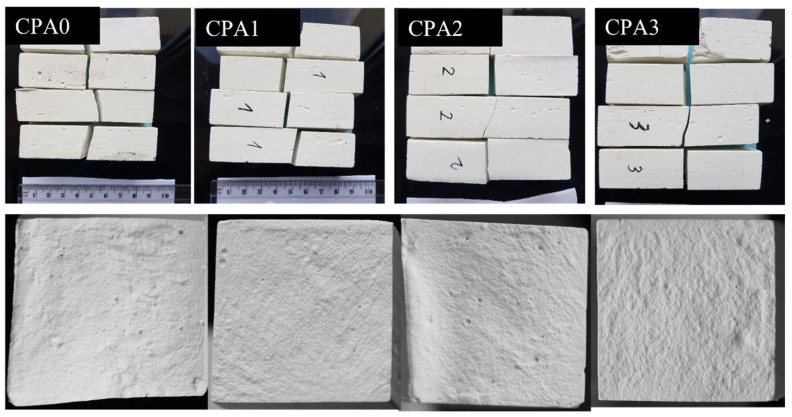
Visual inspection of CPA samples after flexure tests at 28 days, revealing textural changes of the flexure plane associated with variations in accelerator concentration.

New details are obtained by inspecting the fracture interface resulted after the flexure tests, as presented in Figure 5. Notably, the visual inspection of the fracture interface reveals a clear textural change with CN concentration for the entire sample batch: The apparent roughness of the flexure plane increases with CN concentration, and this represents a key factor. The next section is dedicated to related aspects and the correlation between the mechanical properties and structural/micro-structural features of cement-CN composites.

#### 3.4.1. SEM Analysis of Samples Obtained after Flexure Tests

**Figure 6:**
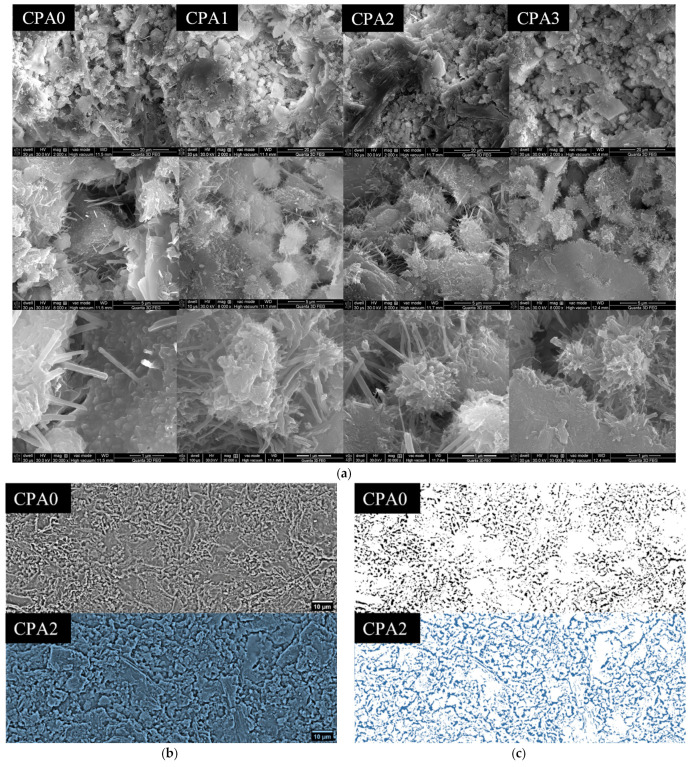
SEM micrographs showing the flexure plane of CPA samples: (**a**) At magnifications of 2000× (scale: 20 µm), 8000× (scale: 5 µm), and 30,000× (scale: 1 µm) for samples CPA samples evidencing the CN-induced changes in capillary pores and in C-S-H/AFt morphology, (**b**) iFFT images and (**c**) binarized images of the panoramic view (2000×, scale: 10 µm) for samples CPA0 (grey) and CPA2 (blue) evidencing the texture obtained after flexure tests.

The SEM analysis on the fractured plane further reveals the changes induced by the accelerator upon the hierarchic assembly of cement, more precisely, upon the microstructure and the morphology of the C-S-H/AFt hydration products. At the microscales (Figure 6a), one can observe aggregates (of the order of 10^0^ µm) with morphologies specific to C-S-H/AFt assemblies. The structures are well evidenced both inside capillary pores (10^1^ µm) and at the interface with the primary cement grains. A first observation is that the morphology of such aggregates seemingly changes with CN concentrations. Following the increase in CN, the exposed needle-like particles, usually associated with AFt, appear to be shorter. This is consistent with the inhibited growth effect induced by CN over AFt as reported in different studies [4]. Further on, the morphology of the C-S-H gel network (with pore dimensions in the range of 10^−1^–10^0^ μm) also appeared to change with CN increase.

In some regions observed at higher magnifications, it appeared that CN may induce an increase in nano-porosity of the C-S-H assembly, which may probably correlate with a larger number of C-S-H dendrites, and a more opened gel morphology (Figure 6a center and bottom row). Even though this observation requires further investigations for statistical support, it is consistent with the increase in fractal size with CN content previously observed by NMR relaxometry [14].

A second aspect concerns the effects on the capillary pore network. To differentiate it and to reduce sampling errors, panoramic views of samples CPA0 and CPA2 were constructed. An image processing sequence was further applied: (1) iFFT bandpass filtering (i.e., between 10, 137 pixels—corresponding to a spatial frequency domain of 0.7, 1 μm, results shown in Figure 6b) and (2) image binarization, where “0” values (black) are associated with pores and “1” values (white), associated with particles and aggregates forming the solid network (results shown in Figure 6c).

The results show that at higher CN concentrations, the flexure interface becomes less compact, and the capillary pore network is formed by broader and more interconnected pores, which supports an increase in fracture roughness with CN content. In other studies, the fracture roughness was shown to correlate with fractographic parameters such as fracture toughness, the effective crack length, and the quasi-brittleness of the material [25]. Ficker et al. [26] analyzed the surface profiles of cementitious systems using confocal optic microscopy and were able to correlate the surface topology parameters (i.e., surface roughness, as height irregularity function) with changes in the w/c ratio, porosity, as well as with the compressive and flexure strengths and further emphasized the main role played by the capillary pore network structure in the parametric description of the surface texture of fracture interfaces [26].

#### 3.4.2. NMR Relaxometry

**Figure 7:**
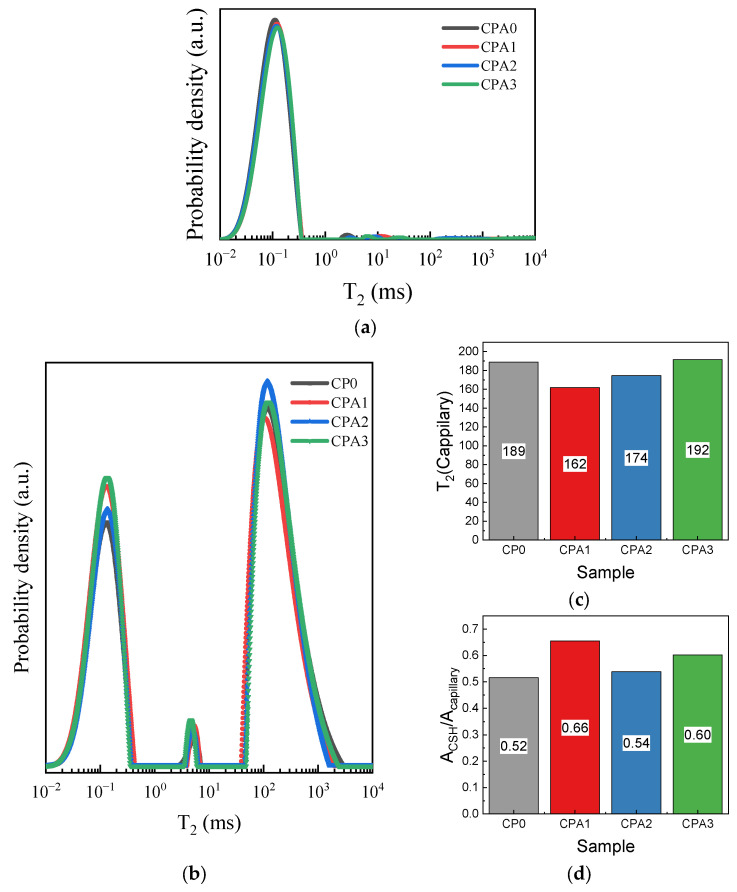
Relaxation time distributions of (**a**) water and (**b**) cyclohexane molecules confined in hardened CPA samples, followed by parameters extracted from the distribution of cyclohexane: (**c**) The center of the T_2_ relaxation peak for capillary pores and (**d**) the ratio between the areas of the intra C-S-H peak (at T_2_~10^−1^ ms) and the capillary peak.

Considering the complexity of the cement-based materials and that spatial distribution of pore sizes at significantly larger scales may induce sampling errors during the SEM analysis, NMR relaxometry experiments were performed on aged CPA samples collected after flexure tests. Initial experiments were performed on the vacuumed samples so that only the water molecules entrapped in the C-S-H lamellae (intra-C-S-H pores with associated dimensions of ~0.5–1.8 nm) or inside the C-S-H pores (inter C-S-H pores of ~2–10 nm) may contribute, as described in earlier studies [15,16,17]. The transverse relaxation time distributions obtained for the dry samples are given in Figure 7a. The large peak centered around T_2_~10^−1^ ms is correlated with water molecules confined both at inter and intra C-S-H sites. It can be observed that the samples modified with CN exhibit a slight shift towards larger relaxation times, probably indicating larger inter C-S-H pore contributions to the relaxation spectra and hence, to the pore size distributions. The smaller contributions with relaxation times found between 10^0^ and 10^2^ ms correspond to partially filled capillary pores.

To avoid the effects of partial saturation described elsewhere [16] and to fully take into account the wider range of pore classes, NMR investigations of samples filled with cyclohexane are further described in Figure 7b. In this case, the intra C-S-H peak maintains its initial position at 10^−1^ ms since water molecules cannot be extracted by cyclohexane from the C-S-H structure [16]. The second peak, ascribed to inter C-S-H pores, is observed between 3 and 9 ms. The area of the inter C-S-H peaks exhibits larger values with CN increase, which further supports the SEM observations that more nano-porous C-S-H gels are obtained in the presence of CN. However, due to the small intensity of the inter C-S-H peak and the ill-posed conditions of the Laplace transform, additional investigations and further statistical support will be required to fully confirm this claim.

The third peak found between 3 × 10^1^ and 3 × 10^3^ ms can be ascribed to capillary pores. Focusing on the correlation between the mechanical properties (Figure 4) and the capillary pore content, some major aspects can be observed. The first observation is that the T_2_ maxima of the capillary peak tend to shift to higher values for CPA1-3 samples (Figure 7c). For the samples collected after flexure tests, T_2_^max^ (CPA1) < T_2_^max^ (CPA0) < T_2_^max^ (CPA3) < T_2_^max^ (CPA2). This is consistent with the increase in the size of the capillary pores observed during the SEM analysis, however, a low Pearson correlation coefficient (−0.42) is found between T_2_^max^ and the flexure strength. Thus, the CN-induced changes to the capillary pore dimensions are insufficient in explaining why CPA3 sample would exhibit improved mechanical properties relative to CPA2 sample. The second observation is based on the significant changes in the capillary peak area with respect to the intra C-S-H peak. When analyzing the intensity ratio of the intra C-S-H to the capillary peak (A_CSH/Capill_), one observes the following trend (Figure 7d): A_CSH/Capill_ (CPA1) > A_CSH/Capill_ (CPA3) > A_CSH/Capill_ (CPA2)~A_CSH/Capill_ (CPA0). This finally results in a high Pearson correlation coefficient of 0.98 found between the A_CSH/Capill_ parameter and the flexural strength.

The NMR data correlate well with the mechanical performance, the SEM, and visual inspection, as well as the structural data obtained through XRD. The results indicate that for the present system based on a commercial white Portland cement, CN may induce two opposing phenomena. First, at nanoscales, the NMR and SEM data indicate that CN may alter the growth patterns of AFt and C-S-H (i.e., due to changes in pH) to ultimately yield a more complex network (i.e., higher fractal dimensions, more opened C-S-H gel networks and higher quantities of CH and C-S-H hydration products). This may yield a higher elastic response with CN increase at early hydration stages. Second, at micro-scales, CN induces an increase in capillary porosity and carbonate yields which is detrimental to the mechanical properties. The competition between the two effects, one dominant at nanoscale, the other at micro-scales, may be responsible for the complex mechanical behavior observed in cement-calcium nitrate composites. An optimal mechanical strength (Figure 4) may be reached at intermediate CN concentrations (i.e., CPA1) when the capillary network is comprised of smaller and less connected pores. At a critical CN concentration (CPA3 at 7 days, CPA2 at 28 days), the mechanical strength decreases as disruptions may occur in the bridging between adjacent nodes, due to the increase in capillary pore volumes. Finally, when densification stages progress beyond 28 days in the presence of higher CN concentrations (when comparing CPA3 with CPA2 systems), a network with higher CH and C-S-H contributions may slightly counteract this effect and lead to a second-order increase in mechanical properties.

## 4. Conclusions

The effects induced by calcium nitrate over white Portland cements were investigated in different fresh cement paste compositions after 7 and 28 days of hardening.

A decrease in slump and setting time is confirmed for samples with CN additions representing 1–3% of the mass of cement. The smaller size of the ettringite needles observed during the SEM investigations and the superplasticizing effect of CN observed during the early hydration stage, i.e., due to promoted (OH)^−^ surface coverage, should be considered when analyzing the slump or flowability of pastes during dormant stages. The flexural and compressive tests on cement samples, with no sand or aggregates added, were performed after hardening at 7 and 28 days. The results indicate a non-monotonous evolution in mechanical strength with CN content. The trends observed in fresh and hardened properties are also consistent with other studies.

Through XRD, SEM, and NMR relaxometry performed on samples after the final flexure tests, several phenomena are revealed: (1) Changes in the morphology of C-S-H gel (2) an increase in the contribution of capillary pores, and (3) an increase in carbonation with CN content for aged samples. The interplay between these phenomena may be reflected by the intra-C-S-H to capillary peak area ratio (A_CSH_/A_capillary_) observed in the T_2_ distribution of NMR spectra. A potential correlation is found between A_CSH_/A_capillary_ and the mechanical properties, which further supports the fact that CN induces changes at both nano- and microscales in the hierarchic design of cement-based materials.

## Data Availability

Not applicable.

## References

[1] Paul S.C., van Zijl G.P.A.G., Tan M.J., Gibson I. (2018). A Review of 3D Concrete Printing Systems and Materials Properties: Current Status and Future Research Prospects. Rapid Prototyp. J..

[2] Valente M., Sibai A., Sambucci M. (2019). Extrusion-Based Additive Manufacturing of Concrete Products: Revolutionizing and Remodeling the Construction Industry. J. Compos. Sci..

[3] Tramontin Souza M., Maia Ferreira I., Guzi de Moraes E., Senff L., Arcaro S., Castro Pessôa J.R., Ribeiro M.J., Novaes de Oliveira A.P. (2022). Role of Chemical Admixtures on 3D Printed Portland Cement: Assessing Rheology and Buildability. Constr. Build. Mater..

[4] Yuan Q., Zhou D., Huang H., Peng J., Yao H. (2020). Structural Build-up, Hydration and Strength Development of Cement-Based Materials with Accelerators. Constr. Build. Mater..

[5] Tao Y., Rahul A.V., Lesage K., Yuan Y., van Tittelboom K., de Schutter G. (2021). Stiffening Control of Cement-Based Materials Using Accelerators in Inline Mixing Processes: Possibilities and Challenges. Cem. Concr. Compos..

[6] Joseph S., Skibsted J., Cizer Ö. (2019). A Quantitative Study of the C3A Hydration. Cem. Concr. Res..

[7] Dorn T., Blask O., Stephan D. (2022). Acceleration of Cement Hydration—A Review of the Working Mechanisms, Effects on Setting Time, and Compressive Strength Development of Accelerating Admixtures. Constr. Build. Mater..

[8] Ramachandran V.S. (1996). Concrete Admixtures Handbook: Properties, Science and Technology.

[9] Kičaitė A., Pundienė I., Skripkiūnas G. (2017). The Influence of Calcium Nitrate on Setting and Hardening Rate of Portland Cement Concrete at Different Temperatures. IOP Conf. Ser. Mater. Sci. Eng..

[10] Skripkiūnas G., Kičaitė A., Justnes H., Pundienė I. (2021). Effect of Calcium Nitrate on the Properties of Portland–Limestone Cement-Based Concrete Cured at Low Temperature. Materials.

[11] Justnes H. (2010). Calcium Nitrate as Multifunctional Concrete Admixture. Alite Inf.–Int. Anal. Rev. Concr. Cem. Dry Admix..

[12] Hill R., Daugherty K. (1996). The Interaction of Calcium Nitrate and a Class C Fly Ash during Hydration. Cem. Concr. Res..

[13] Justnes H., Nygaard E. (1999). Calcium Nitrate—A Multifunctional Admixture For High Performance Concrete. Int. RILEM Conf..

[14] Ardelean I. (2021). The Effect of an Accelerator on Cement Paste Capillary Pores: NMR Relaxometry Investigations. Molecules.

[15] Rusu M.M., Vilau C., Dudescu C., Pascuta P., Popa F., Ardelean I. (2023). Characterization of the Influence of an Accelerator upon the Porosity and Strength of Cement Paste by Nuclear Magnetic Resonance (NMR) Relaxometry. Anal. Lett..

[16] Bede A., Scurtu A., Ardelean I. (2016). NMR Relaxation of Molecules Confined inside the Cement Paste Pores under Partially Saturated Conditions. Cem. Concr. Res..

[17] Bede A., Ardelean I. (2017). Revealing the Influence of Water-Cement Ratio on the Pore Size Distribution in Hydrated Cement Paste by Using Cyclohexane. AIP Conf. Proc..

[18] Tan Z., Bernal S.A., Provis J.L. (2017). Reproducible Mini-Slump Test Procedure for Measuring the Yield Stress of Cementitious Pastes. Mater. Struct..

[19] Schindelin J., Arganda-Carreras I., Frise E., Kaynig V., Longair M., Pietzsch T., Preibisch S., Rueden C., Saalfeld S., Schmid B. (2012). Fiji: An Open-Source Platform for Biological-Image Analysis. Nat. Methods.

[20] Lutterotti L., Matthies S., Wenk H.-R., Schultz A.S., Richardson J.W. (1997). Combined Texture and Structure Analysis of Deformed Limestone from Time-of-Flight Neutron Diffraction Spectra. J. Appl. Phys..

[21] Meiboom S., Gill D. (1958). Modified Spin-Echo Method for Measuring Nuclear Relaxation Times. Rev. Sci. Instrum..

[22] Venkataramanan L., Song Y.Q., Hurlimann M.D. (2002). Solving Fredholm Integrals of the First Kind with Tensor Product Structure in 2 and 2.5 Dimensions. IEEE Trans. Signal Process..

[23] Dorn T., Hirsch T., Stephan D. (2019). Study on the Influence of Accelerators on the Hydration of Portland Cement and Their Applicability in 3D Printing. RILEM.

[24] Scrivener K.L., Füllmann T., Gallucci E., Walenta G., Bermejo E. (2004). Quantitative Study of Portland Cement Hydration by X-Ray Diffraction/Rietveld Analysis and Independent Methods. Cem. Concr. Res..

[25] Lange D.A., Jennings H.M., Shah S.P. (1993). Relationship between Fracture Surface Roughness and Fracture Behavior of Cement Paste and Mortar. J. Am. Ceram. Soc..

[26] Ficker T. (2017). Rupture Strength and Irregularity of Fracture Surfaces. IOP Conference Series: Materials Science and Engineering.

